# The Influence of High-Power Ultrasound and Bactofugation on Microbiological Quality of Milk

**DOI:** 10.17113/ftb.59.04.21.7179

**Published:** 2021-12

**Authors:** Edita Juraga, Višnja Stulić, Tomislava Vukušić Pavičić, Jasenka Gajdoš Kljusurić, Mladen Brnčić, Zoran Herceg

**Affiliations:** 1 ATERA d.o.o., Ivane Brlić Mažuranić 25, 42000 Varaždin, Croatia; 2 Faculty of Food Technology and Biotechnology, University of Zagreb, Pierottjeva 6, 10000 Zagreb, Croatia

**Keywords:** high-power ultrasound, microbiological safety of milk, technology of bactofugation

## Abstract

**Research background:**

The application of high power ultrasound combined with a slightly increased temperature on raw cow’s milk, skimmed cow’s milk and skimmed cow’s milk that passed the bactofugation process was analysed. We combined ultrasound with bactofugation of milk to achieve the microbiological accuracy that is equivalent to pasteurization.

**Experimental approach:**

The milk samples (200 mL) were treated for 2.5, 5, 7.5 and 10 min with high-power ultrasound (200 and 400 W) with a frequency of 24 kHz. The treatments were conducted with a constant duty cycle of 100%. Temperatures during the treatments were 20 and 55 °C. The somatic cell count of the aerobic mesophilic bacteria, as well the number of *Enterobacteriaceae, Escherichia coli* and *Staphylococcus aureus* cells were analysed.

**Results and conclusions:**

From the perspective of the reduction of the total count of bacteria, the best result was achieved by high-power ultrasound at 400 W treated for 10 min. High reduction of *Enterobacteriaceae*, *E. coli* and *S. aureus* cells was achieved with ultrasound treatment of raw, skimmed and skimmed cow’s milk that passed the bactofugation with a power of 200 and 400 W regardless of the treatment time.

**Novelty and scientific contribution:**

This work combines bactofugation and high-power ultrasound for the inactivation of microoganisms. This combination was used at a slightly increased temperature (up to 55 °C), which is much more economical than pasteurization, while it preserves the sensory and physicochemical properties of milk.

## INTRODUCTION

Milk is a biological fluid that deserves special attention as the most complete natural fluid (*1*). It is an ideal medium for the development of undesirable microorganisms (*2*). To ensure the safety of food, raw milk needs to be controlled by conducting chemical and microbiological analyses which determine its quality. If the microbiological analysis reveals more than 10^5^ CFU/mL microorganisms in raw milk, the result indicates a lack of hygienic conditions (*3*), and the somatic cell count (SCC) in 1 mL must be ≤400 000, observed as a geometric average over three months.

The Food and Agriculture Organization of the United Nations and the World Health Organization define various thermal procedures that are carried out to reduce and remove the number of microorganisms from milk (*4*). The most common processes are continuous flow pasteurization and ultra-high temperature (UHT) treatment. However, significant effects of heat treatment of milk are vitamin degradation, whey protein denaturation and Maillard reaction (due to protein and lactose reaction). Therefore, it is extremely important to use lower temperatures with the same or higher efficiency as pasteurization and/or sterilization.

Bactofugation is used to improve the bacteriological quality of raw milk. It belongs to mechanical processes, and is used in the production of pasteurized and UHT milk. This process reduces the primary number of heat-resistant microorganisms in milk before heat treatment, all to prolong the shelf life of milk using a milder temperature regime (*5*). The optimal temperature of bactofugation, at which the best results are achieved, is 55–60 °C.

Today, non-thermal methods as the high-power ultrasound (*6*, *7*) treatment with high hydrostatic pressure, pulsed electric and magnetic fields are often used in food industry. High hydrostatic pressure is commercially applied in food processing, and ultrasound is applied in homogenization, emulsification and dispersion processes (*8*). New non-thermal methods can significantly save energy and shorten the duration of the production. The use of high-power ultrasound has shown several advantages over heat treatment by pasteurization, such as minimizing taste loss in juices, greater homogeneity and significant energy savings (*9*). During the high-power ultrasound processing, acoustic energy transfer is instantaneous and extends through the entire volume, which results in lower energy consumption (*6*). When using low-intensity ultrasound waves, the mechanism of microorganism inactivation is based on changing the metabolism of microorganism cells, while the mechanism of microorganism inactivation using high-power ultrasound waves is based on breaking cell membranes of microorganisms and denaturation of enzymes (*10*, *11*).

Therefore, the aim of this paper is to examine the possibility of processing raw and skimmed milk using high-power ultrasound in combination with slightly elevated temperature and pretreated with bactofugation in order to achieve microbiological safety at the level achieved by pasteurization.

## MATERIALS AND METHODS

### Milk samples

Throughout the research, milk at different stages of processing was used, sampled directly from production, where bactofugation is an integral part of the milk processing.

All tests were performed on cow's milk from the same production batch from the dairy Vindija plc (Varaždin, Croatia). The tests were performed on raw, skimmed and skimmed bactofuged milk, and on pasteurized milk as a reference sample. Milk samples were aseptically taken into sterile vials at the sampling valves before the separator (raw milk), after the separator (skimmed milk), and after the bactofugation (skimmed bactofuged milk). As a reference control sample, pasteurized milk produced by the classical HTST (high-temperature short-time) process (processing parameters 72 °C/15 s) was taken.

### Ultrasonic processing of milk samples

In this research, an ultrasonic processor model UP 400S (Dr. Hielscher GmbH, Teltow, Germany) was used. The characteristics of this ultrasonic processor are opened system with an effective output power 400 W, current voltage 230 V, 48-63 Hz, ultrasonic cycle 10–100%, ultrasonic frequency 24 kHz and amplitude 12-260 μm. A 7-mm diameter titanium probe was used in the work, and it was immersed at a depth of 2 cm in each milk sample.

### Determination of milk somatic cell count

The count of somatic cells in milk, epithelial cells and leukocytes was determined by electronic cell counter (Fossomatic 5000; Foss Electric A/S, Hillerød, Denmark) using a cytometric method of fluoro-optoelectronic counting according to ISO 13366-2:2006 standard (*12*).

### Determination of hygienic quality of milk

To determine the inactivation of the tested microorganisms before the treatment of the raw, skimmed and bactofuged milk samples, the initial number of tested microorganisms was determined. The reduction of the logarithmic number of microorganism cells after treatment was calculated according to the formula:



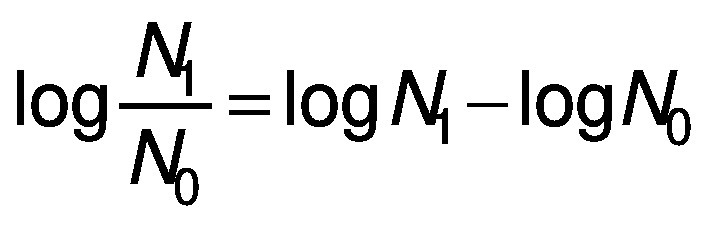



where *N*_0_ is the total initial number of microorganisms before and *N*_1_ after the treatment.

### Design of the experiment

The design of the experiment was marked with letters from A to D: (*i*) experiment A: *P*_ultrasound_=200 W, *ν*=24 kHz, *t*=20 °C, (*ii*) experiment B: *P*_ultrasound_=200 W, *ν*=24 kHz, *t*=55 °C, (*iii*) experiment C: *P*_ultrasound_=400 W, *ν*=24 kHz, *t*=20 °C, and (*iv*) experiment D: *P*_ultrasound_=400 W, *ν*=24 kHz, *t*=55 °C.

Treatments were performed at four different times (2.5, 5, 7.5 and 10 min). Raw, skimmed and bactofuged cow's milk were subjected to high-power ultrasound treatment of 200 and 400 W. Reductions of SCC, *Enterobacteriaceae*, *E. coli* and S. *aureus* were calculated. Each sample was analyzed three times and the presented results are the mean value of three measurements.

### Preparation of samples

A volume of 1 mL of prepared decimal dilutions of samples was added to Petri dishes and poured over with a liquid nutrient medium. Furthermore, the samples were also inoculated on a prepared solid medium. A volume of 0.1 mL of a milk sample was added, and smeared with a Drigalsky stick. The samples were incubated according to the ISO 4833-1:2013 standard (*13*). Aerobic mesophilic bacteria were incubated at (30±1) °C for (72±2) h.

Incubation of *Enterobacteriaceae* was done at (37±1) °C for (24±2) h. *Enterobacteriaceae* were inoculated on a VRBG agar (crystal violet neutral red bille glucose agar, Merck, Darmstadt, Germany) and then coated with a cover layer of VRBG agar (15 mL) which, after solidification, prevented colony overgrowth and provided semi-anaerobic conditions (*14*).

*Escherichia coli* cells were analyzed on a TBX agar (tryptone bile X-glucuronide agar, Chromocult, Merck). Incubation was performed at (44±1) °C for (21±3) h (*15*).

*S. aureus* cells were confirmed by inoculating the samples on Baird-Parker agar (Merck) with the addition of egg yolk and tellurite emulsion. The cells were incubated at (37±1) °C for (24±2) h. When colonies appeared on the medium, they were confirmed by a positive catalase test. The test was performed using Bactident catalase reagent (Merck). The procedure was performed in such a way that a drop of reagent was added directly to a randomly selected colony on the medium. *S. aureus* colonies produce gas (*16*). If *S. aureus* colonies were not confirmed in the result section, they were indicated as not found (n.f.). If the count of *S. aureus* cells was less than 10 colonies, it was marked as <10, and if the number of cells was less than 100, it was marked as <100. The same was applied for the determination of *Enterobacteriaceae* and *E. coli*. If the number of cells was higher than 100, the exact number of colonies was specified.

### Determination of the total viable count of bacteria

The total viable count (TVC) of bacteria was determined based on the number of counted colonies multiplied by the degree of dilution (*17*). Increased colonies were counted on a counter (colony counter, Kinesis Ltd, Saint Neots, UK), and the number of viable bacteria in the processed samples was expressed as the colony forming units (CFU). The number of units forming colonies was calculated according to the following formula and ISO 13366-2:2006 standard (*12*):



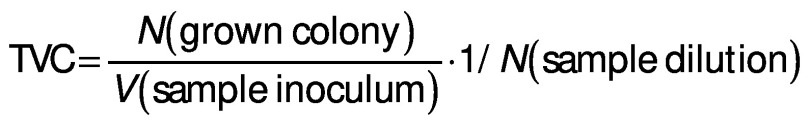



### Statistical data processing

Descriptive statistics was used to show mean values, standard deviation (S.D.), and minimum and maximum values for each experiment (*18*). In order to connect, *i.e*. determine the similarities and/or differences in a large data set for each observed characteristic or treatment, according to the experiment, multivariate statistical methods were applied (*19*). STATISTICA data analysis software v. 8 was used for data processing (*20*).

## RESULTS AND DISCUSSION

The average values ​​of the somatic cell count (SCC) and their reduction in different experiments (A-D) are shown in Table 1. Lower count of somatic cells in untreated skimmed milk (SO) samples than in raw milk (RM) samples was observed. This is explained by the fact that the milk was collected in a centrifugal cream separator, and that part of the somatic cells ends up in the cream. To make it easier to monitor the impact of a treatment on the SCC, a reduction expressed in percentages was calculated. This means that the initial SCC from the reference sample of raw milk (RM) was used to recalculate the T1–T4 treatment reduction, and the skimmed milk (SO) sample was used to recalculate the T5–T12 treatment reduction. Considering the used bactofugation technique, the decrease of the somatic cell count in the reference sample of bactofuged skimmed milk (BSM) was noticeable in comparison with the reference sample of untreated skimmed milk (SO). Besides the fact that bactofugation removed 80-90% bacteria and 90-95% spores (*1*), it is evident that it also removes somatic cells.

**Table 1:** Influenece of different experimental conditions on the somatic cell count (SCC) in various milk samples and their reduction with time

Sample	Treatment							
A		B		C		D	
SCC/(cell/mL)	SCC_reduction_/%	SCC /(cell/mL)	SCC_reduction_/%	SCC/(cell/mL)	SCC_reduction_/%	SCC/(cell/mL)	SCC _reduction_/%
RM	320 000	0	365 000	0	278 000	0	347 000	0
T1	227 000	29	329 000	10	36 000	87	178 000	49
T2	132 000	59	280 000	23	19 000	93	110 000	68
T3	134 000	58	130 000	64	17 000	94	54 000	84
T4	84 000	74	160 000	56	14 000	95	63 000	82
SO	262 000	0	192 000	0	264 000	0	201 000	0
T5	130 000	50	120 000	38	119 000	55	140 000	30
T6	50 000	81	95 000	51	67 000	75	106 000	47
T7	37 000	86	84 000	56	37 000	86	68 000	66
T8	21 000	92	76 000	60	21 000	92	24 000	88
BSM	19 000	93	17 000	91	9 000	97	24 000	88
T9	11 000	96	16 000	92	7 000	97	10 000	95
T10	8 000	97	12 000	94	4 000	98	7 000	97
T11	6 000	98	10 000	95	2 000	99	4 000	98
T12	4 000	98	8 000	96	2 000	99	3 000	99
PM	22 000	92	24 000	88	7 000	97	18 000	91

Experiments A and B: *P*=200 W, *ν*=24 Hz, *t*=20 and 55 °C, respectively. Experiments C and D: *P*=400 W, *ν*=24 Hz, *t*=20 and 55 °C, respectively. Reference samples for calculating the reduction of SCC: RMF=raw milk, SO=untreated skimmed milk, BSM=bactofuged skimmed milk, PM=pasteurized milk. T1–T4=raw milk, T5–T8=skimmed milk and T9–T12=bactofuged skimmed milk treated for 2.5, 5, 7.5 and 10 min

**Fig. 1:**
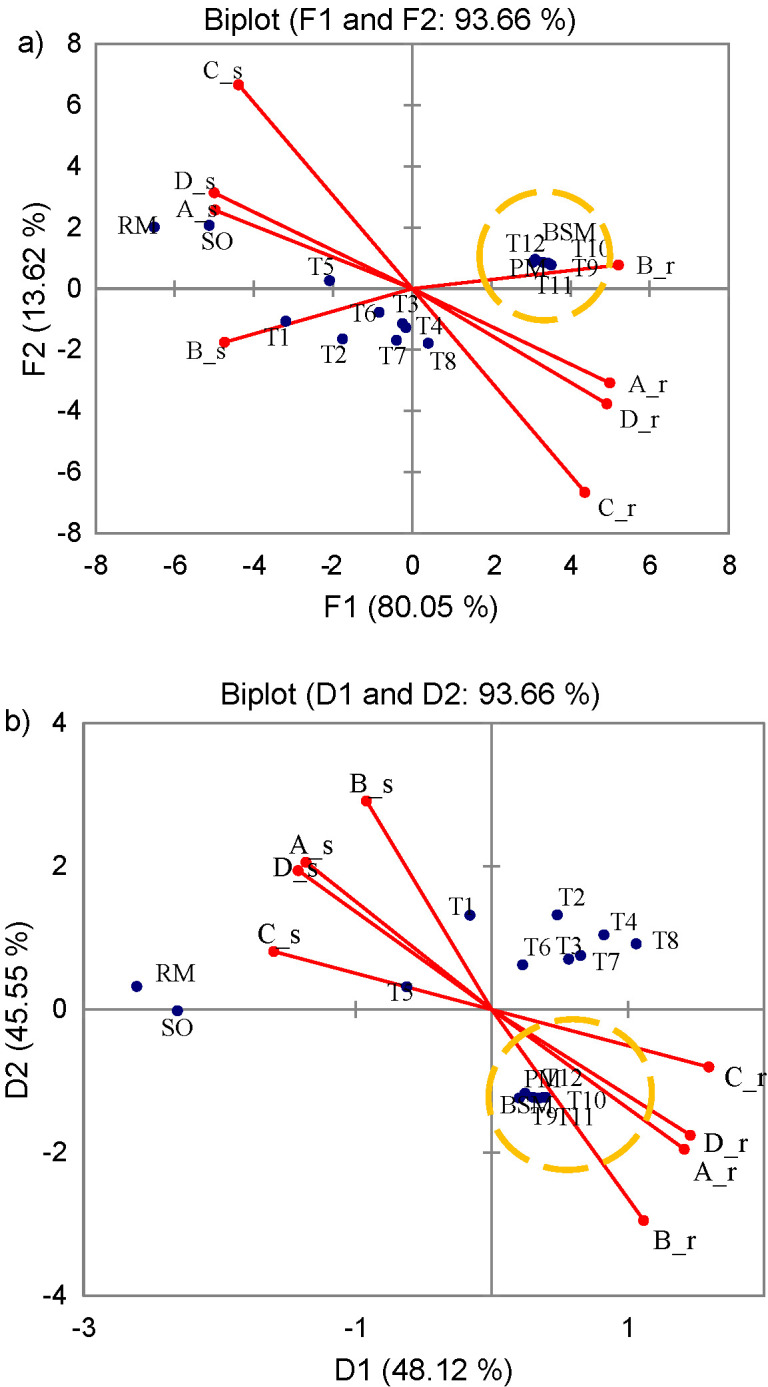
Principal component analysis of somatic cell distribution (SCC), and their reduction: a) without rotation, and b) after Verimax rotation. Experiments A and B: *P*=200 W, *ν*=24 Hz, *t*=20 and 55 °C, respectively. Experiments C and D: *P*=400 W, *ν*=24 Hz, *t*=20 and 55 °C, respectively. Reference samples: RM=raw milk, SO=untreated skimmed milk, BSM=bactofuged skimmed milk, PM=pasteurized milk. T1–T4=raw milk, T5–T8=skimmed milk and T9–T12=bactofuged skimmed milk treated for 2.5, 5, 7.5 and 10 min

Experiments A-D have shown that high-power ultrasound, regardless of temperature used in the treatment, reduces the somatic cell count by creating high local temperature and pressure that cause cell wall rupture and cell disintegration (*21*, *22*). In all experiments, it was observed that the reduction of SCC was higher in ultrasound-treated samples (even at 2.5 min) than in pasteurized samples (in the pasteurization process, bactofugation was included as part of the process (Fig. 1a)). This is in agreement with the findings of Povey and Mason (*23*) and Cameron (*24*), who reported that ultrasound treatment of milk significantly reduces the count of somatic cells. However, reduction of somatic cell count did not improve milk quality due to high initial number of somatic cells.

For each experiment, the observed relationship of the pooled data (Fig. 1) with the corresponding Pearson correlation matrix (Table 2) is shown. The results show the expected negative correlation between the somatic cell count and their reduction, which implies their inversely proportional relationship depending on the experimental conditions (A-D). Grouping of bactofuged samples was seen in all experiments; in Fig. 1a in the first and in Fig. 1b in the fourth quadrant. The analysis of the main components of the somatic cell count distribution and its reduction before rotation is shown in Fig. 1a. Treated milk samples (T8–T12, BSM and PM) were grouped on the right side of the plot, representing samples in which significantly higher reduction of SCC was achieved than in the samples located on the left side of the plot (T1–T7, RM and SO). Bactofuged samples (T9–T12, BSM and PM) were positioned in the first quadrant and correlated with the results of SCC reduction from all experiments (A-D; 1st and 4th quadrant). The contribution of the first and second components is also important (F1=80.05%, F2=13.62%), and the dominance of the first component is visible. Precisely for this reason, rotation was used to distribute the influence of the principal components. Verimax rotation, which is often used in the food industry, was applied and the results are shown in Fig. 1b. It was observed that the share of variations explained in this data set after the rotation remains the same with a high 93.67%, and the bactofugated samples, as shown in Fig. 1b, grouped separately, but now the reduction data of SCC in different experiments no longer spread through the 1st and 4th quadrants. They were grouped only in the 4th quadrant, and the SCC, depending on the experiments, was grouped in the 2nd quadrant. As expected, the ratio of SCC in the milk and its reduction was inversely proportional (Fig. 1b). The first main component explains the high 48% variation in the SCC and its reduction in experiments A, C and D with better reduction results, while the second main component explains the variations in less successful experiments B in terms of total SCC reduction (T1–T12, SO, BSM and PM).

**Table 2:** Pearson correlation matrix for somatic cell count (SCC) and its reduction, depending on the treatment, with a significance level of 5%

Observed	SCC/(cell/mL) (_s)				SCC_reduction_/% (_r)			
A_s	B_s	C_s	D_s	A_r	B_r	C_r	D_r
A_s	1.00	0.90	0.82	0.94	-0.99	-0.92	-0.81	-0.87
B_s	0.90	1.00	0.58	0.87	-0.85	-0.92	-0.57	-0.72
C_s	0.82	0.58	1.00	0.88	-0.86	-0.75	-1.00	-0.93
D_s	0.94	0.87	0.88	1.00	-0.93	-0.90	-0.87	-0.93
A_r	-0.99	-0.85	-0.86	-0.93	1.00	0.92	0.86	0.91
B_r	-0.92	-0.92	-0.75	-0.90	0.92	1.00	0.75	0.89
C_r	-0.81	-0.57	-1.00	-0.87	0.86	0.75	1.00	0.93
D_r	-0.87	-0.72	-0.93	-0.93	0.91	0.89	0.93	1.00

Experiments A and B: *P*=200 W, *ν*=24 Hz, *t*=20 and 55 °C, respectively. Experiments C and D: *P*=400 W, *ν*=24 Hz, *t*=20 and 55 °C, respectively. _s=somatic cells, _r=reduction of somatic cells

Correspondence analysis (CA) is a method of data visualization that is applicable to contingency tables (*25*). It was performed with the aim of comparing differently treated samples in different experiments and the efficiency of SCC reduction. Table 3 shows the change in the SCC, its significance and representation in the first two factors (F1 and F2). In a bactofuged sample treated for 10 min (T12), the SCC count ranged from 2000, in experiment C, to 8000 cell/mL, in experiment B. In Table 3, when observing SCC, there are two columns showing the representation in the reduced number of factors (reduced to two, *i.e*. F1 and F2) and the RM sample dominates in the factor F1, and T1 and T2 in the second factor, F2. Their dominance in factor F2 was associated with a high somatic cell count.

**Table 3:** Reduction of somatic cell count (SCC), its significance and representation in the first two factors for different treatments

Milk sample	SCC/(cell/mL)			SCC_reduction_/%		
Significant order	F1	F2	Significant order	F1	F2
RM	1	0.275	0.010	15	0.000	0.000
T1	3	0.001	0.283	14	0.098	0.521
T2	4	0.043	0.162	12	0.105	0.123
T3	7	0.050	0.017	7	0.029	0.007
T4	6	0.101	0.010	9	0.073	0.001
SO	2	0.230	0.097	15	0.000	0.000
T5	5	0.013	0.071	13	0.029	0.052
T6	8	0.017	0.099	11	0.079	0.148
T7	9	0.075	0.105	10	0.089	0.084
T8	10	0.173	0.124	8	0.118	0.062
BSM	12	0.011	0.004	5	0.068	0.000
T9	13	0.001	0.001	4	0.064	0.000
T10	14	0.000	0.002	3	0.064	0.000
T11	15	0.001	0.001	2	0.062	0.000
T12	16	0.004	0.001	1	0.061	0.000
PM	11	0.003	0.012	6	0.063	0.003

Reference samples for calculating the reduction of SCC: RM=raw milk, SO=untreated skimmed milk, BSM=bactofuged skimmed milk, PM=pasteurized milk. T1–T4=raw milk, T5–T8=skimmed milk and T9–T12=bactofuged skimmed milk treated for 2.5, 5, 7.5 and 10 min

The antimicrobial effect of ultrasound is achieved by cavitation, *i.e*. extremely rapid formation and collapse of bubbles formed in the medium by the action of ultrasonic waves. This effect occurrs due to changes in pressure and temperature, which cause cell wall rupture and thinning of the cell membrane (*22*, *26*, *27*). Also, due to the action of free radicals, DNA damage can occur. Table 4 shows the total viable count (TVC) of bacteria and the reduction of their logarithmic number (LNC) in milk samples according to different conditions of experiments A–D. Analysis of LNC reductions within the same experiment (regardless of power, frequency and temperature parameters) shows that the best results were achieved by ultrasound treatment of bactofuged skimmed milk samples in the follwing order: experiment D (reduction of LNC from T9=2.31 to T12=2.68), experiment B (reduction from T9=2.14 to T11=2.44), experiment C (reduction from T12=2.06 to T9/10=2.20) and experiment A (reduction from T10=1.94 to T12=2.04). From the analyzed data, it can be concluded that better results of TVC reduction were observed in experiments D (*P*=400 W, *t*=55 °C) and B (*P*=200 W, *t*=55 °C), in which milk was treated by high-power ultrasound. Cameron (*24*) stated that in order to achieve better results for the inactivation of microorganisms in milk, it is recommended to combine high-power ultrasound with slightly elevated temperature, which we confirmed in this work. Many other authors discussed that the inactivation of microorganisms exposed to the combination of ultrasound and temperature is much higher, which is consistent with the results of this work (*28*, *29*). According the regulations from the Rules on microbiological criteria (*30*) (m=10^3^ CFU/mL, M=10^4^ CFU/mL, n=5, c=1, where m is the limit value below which all results are considered satisfactory, and M is the limit value above which the results are considered unsatisfactory), only the results of the number of bacteria in bactofuged samples of A–D experiments and pasteurized milk reference samples (PM) proved to be satisfactory. Table 5 shows the contribution of experiments A–D, their significance and representation in the first two factors for the changes in total number of bacteria (CFU/mL).

**Table 4:** Influence of different experimental treatments on total number of bacteria and reduction of logarithmic number in milk samples

Milk sample		Treatment										
A			B			C			D		
TVC	LNC	LNC_reduction_	TVC	LNC	LNC_reduction_	TVC	LNC	LC_reduction_	TVC	LNC	LNC_reduction_
CFU/mL	log CFU/mL		CFU/mL	log CFU/mL		CFU/mL	log CFU/mL		CFU/mL	log CFU/mL	
RM	450 000	5.65	0.00	1 050 000	6.02	0.00	400 000	5.60	0.00	580 000	5.76	0
T1	110 000	5.04	0.61	142 000	5.15	0.87	72 000	4.86	0.75	31 000	4.49	1.27
T2	57 600	4.76	0.89	126 000	5.10	0.92	40 000	4.60	1.00	18 000	4.26	1.51
T3	48 000	4.68	0.97	24 000	4.38	1.64	25 000	4.40	1.20	4 000	3.60	2.16
T4	76 800	4.89	0.77	16 000	4.20	1.82	44 000	4.64	0.96	5 000	3.70	2.06
SO	438 000	5.64	0.00	656 000	5.82	0.00	160 000	5.20	0.00	408 000	5.61	0.00
T5	69 000	4.84	0.80	100 000	5.00	0.82	32 000	4.51	0.70	78 000	4.89	0.72
T6	100 000	5.00	0.64	120 000	5.08	0.74	23 000	4.36	0.84	58 000	4.76	0.85
T7	96 000	4.98	0.66	140 000	5.15	0.67	29 000	4.46	0.74	60 000	4.78	0.83
T8	62 000	4.79	0.85	29 000	4.46	1.36	20 000	4.30	0.90	8 800	3.94	1.67
BSM	11 000	4.04	1.60	17 800	4.25	1.57	4 080	3.61	1.59	11 000	4.04	1.57
T9	4 560	3.66	1.98	4 800	3.68	2.14	1 000	3.00	2.20	2 000	3.30	2.31
T10	5 000	3.70	1.94	4 200	3.62	2.19	1 000	3.00	2.20	1 100	3.04	2.57
T11	4 160	3.62	2.02	2 400	3.38	2.44	1 200	3.08	2.13	1 300	3.11	2.50
T12	4 000	3.60	2.04	2 600	3.42	2.04	1 400	3.15	2.06	860	2.93	2.68
PM	336	2.53	3.12	100	2	3.82	100	2.00	3.20	920	2.96	2.65

Experiments A and B: *P*=200 W, *ν*=24 Hz, *t*=20 and 55 °C, respectively. Experiments C and D: *P*=400 W, *ν*=24 Hz, *t*=20 and 55 °C, respectively. Reference samples for calculating the reduction of bacterial count: RM=raw milk, SO=untreated skimmed milk, BSM=bactofuged skimmed milk, PM=pasteurized milk. T1–T4=raw milk, T5–T8=skimmed milk and T9–T12=bactofuged skimmed milk treated for 2.5, 5, 7.5 and 10 min. TVC=total viable count of bacteria, LNC=logarithm of total count of bacteria

**Table 5:** Contribution of experiments A-D, their significance and representation in the first two factors for the changes in total number of bacteria

Experiment	Significance	F1	F2
A	4	0.001	0.031
B	3	0.339	0.013
C	6	0.031	0.235
D	5	0.272	0.059

Experiments A and B: *P*=200 W, *ν*=24 Hz, *t*=20 and 55 °C, respectively. Experiments C and D: *P*=400 W, *ν*=24 Hz, *t*=20 and 55 °C, respectively

In accordance with the first part of Singh and Heldman's assumption (*31*), the combination of ultrasound and heat should result in a product with a longer shelf life, and the required processing time could even be reduced, leading to lower production cost. The main problem here, however, is the required processing time with ultrasound, which is much longer than the classical pasteurization method (7.5–10 min *versus* 0.25 min). One of the possibilities to solve this problem is to install ultrasonic probes in the process after bactofugation.

Herceg *et al*. (*32*) noted that the system of high-power ultrasound processing of milk in industrial production should be designed to allow maximum contact between the milk and the cavitation zone, and that it would be useful to explore the possibility of using multiple ultrasound probes. The action of a parallel series of ultrasound probes should be further demonstrated and confirmed. This would be in line with the suggestion by Ashokkumar *et al*. (*33*), who explained that in the dairy industry, it would be interesting to add ultrasound as a new process function to improve the functionality of the products. Furthermore, Oliveira and Oliveirana (*34*) represented in their work that higher inactivation of microorganisms was obtained when using the sonication technique at 70 °C. Results achieved by Sala *et al*. (*35*) in the inactivation of microorganisms using thermal sonication of milk at 70 °C, which is also used in standard high-temperature short-time pasteurization (HTST) procedure, showed high reduction results. They consider that milk treated in this way would not contain vegetative cells, and that it would have a longer shelf life with minimal processing. Furthermore, the product could be similar, in terms of microorganism content, to ultra-high temperature (UHT) milk.

It is prescribed that the number of *Enterobacteriaceae* must be within the following limits m=M≤10 CFU/mL for the sample to be considered satisfactory. In the results shown in Table 6, it was observed that high-power ultrasound treatment of milk gives good results of *Enterobacteriaceae* inactivation. Satisfactory results of the treatment of raw milk T1–T4 samples were observed in high-power ultrasound experiments B and D, where the power was 200 and 400 W, respectively, combined with an elevated temperature of 55 °C. The same trend was observed in the samples T5–T8 of the same experiments (B and D), and F.

**Table 6:** Influence of treatments on total number of *Enterobacteriaceae* and number of *Escherichia coli* cells

Milk sample	Treatment							
A	B	C	D	A	B	C	D
*N*(*Enterobacteriaceae*)/(CFU/mL)				*N*(*Escherichia coli*)/(CFU/mL)			
RM	900	510	200	910	500	390	210	730
T1	840	10	140	<10	410	10	90	<10
T2	720	<10	110	<10	100	<10	100	<10
T3	1 000	<10	140	<10	500	<10	50	<10
T4	360	<10	160	<10	<100	<10	20	<10
SO	200	200	130	620	180	110	100	100
T5	100	n.n.	<100	<10	100	<10	<100	<10
T6	<100	<10	<100	<10	<100	<10	<100	<10
T7	<100	<10	<100	<10	100	<10	<100	<10
T8	<100	<10	<100	<10	<100	<10	<100	<10
BSM	<10	<10	15	20	<10	<10	13	10
T9	<10	<10	10	<10	<10	<10	10	<10
T10	<10	<10	10	<10	<10	<10	10	<10
T11	<10	<10	<10	<10	<10	<10	<10	<10
T12	<10	<10	10	<10	<10	<10	10	<10
PM	<10	<10	<10	<10	<10	<10	<10	<10

Experiments A and B: *P*=200 W, *ν*=24 Hz, *t*=20 and 55 °C, respectively. Experiments C and D: *P*=400 W, *ν*=24 Hz, *t*=20 and 55 °C, respectively. Reference samples: RM=raw milk, SO=untreated skimmed milk, BSM=bactofuged skimmed milk, PM=pasteurized milk. T1–T4=raw milk, T5–T8=skimmed milk and T9–T12=bactofuged skimmed milk treated for 2.5, 5, 7.5 and 10 min

The number of *Escherichia coli* in milk (Table 6) was also observed. Cameron *et al.* (*24*) suggested that ultrasound cavitation destroys the cells of undesirable contaminants such as *E. coli* bacteria, which is confirmed by the results in this work. As with *Enterobacteriaceae*, satisfactory results of treatment of raw milk T1–T4 samples were observed in high-power ultrasound treated samples in experiments B and D, and in samples F2 (5 min) and F4 (10 min). The same trend was observed in samples T5–T8 in experiments B and D.

Table 7 shows the results of *Staphylococcus aureus* inactivation in all experiments, with satisfactory results of T1–T4 whole milk treatments in all high-power ultrasound samples of experiments B, D and F. The same trend is seen in high-power ultrasound samples T5–T8 in experiments B and D. In all experiments with reference samples BSM and PM, and treatments T9–T12, it was observed that the *S. aureus* number met the criterion M=10 CFU/mL. It can be seen that in samples B9/10, D9/10, F9–F12 and P (experiments B and D) the presence of bacteria was not proven/found (n.f.), which means that these samples were additionally investigated to confirm the presence of *S. aureus* species. Oliveira and Oliveirana (*34*) investigated the inactivation of *S. aureus* cells by high-power ultrasound treatment and concluded that higher inactivation was obtained when using a combination of ultrasound with slightly elevated temperature than when using only ultrasound for inactivation, which is in accordance with the results obtained in this work. Sherba *et al.* (*36*) studied the effect of ultrasound (24 kHz) on *S. aureus* species and concluded that it has a bactericidal effect, and that inactivation of this bacteria increases with time and intensity of ultrasound. Thus, the reduction of *S. aureus* in their work increased from 22 to 39% with an increase in the intensity from 1 to 3 for 15 min, or with an extension of the ultrasound time (2-30 min, 3 W/cm^2^), the reduction was 42-43%.

**Table 7:** Influence of treatments on total number of *Staphylococcus aureus* cells

Milk sample		Treatment		
A	B	C	D
*N*(*Staphylococcus aureus*)/(CFU/mL)			
RM	200	350	150	100
T1	160	10	180	<10
T2	170	n.f.	120	<10
T3	110	<10	120	<10
T4	140	<10	110	<10
SO	120	130	<100	100
T5	<100	n.f.	<100	<10
T6	<100	n.f.	<100	n.f.
T7	<100	<10	100	<10
T8	<100	<10	<100	<10
BSM	<10	<10	<10	<10
T9	10	n.f.	<10	n.f.
T10	<10	n.f.	<10	n.f.
T11	<10	<10	<10	<10
T12	<10	<10	<10	<10
PM	<10	n.f.	<10	n.f.

Experiments A and B: *P*=200 W, *ν*=24 Hz, *t*=20 and 55 °C, respectively. Experiments C and D: *P*=400 W, *ν*=24 Hz, *t*=20 and 55 °C, respectively. Reference samples: RM=raw milk, SO=untreated skimmed milk, BSM=bactofuged skimmed milk, PM=pasteurized milk. n.f.=not found. T1–T4=raw milk, T5–T8=skimmed milk and T9–T12=bactofuged skimmed milk treated for 2.5, 5, 7.5 and 10 min

## CONCLUSIONS

This work deals with the possibility of processing raw and skimmed cow's milk using high-power ultrasound in combination with slightly elevated temperature and pretreatment with bactofugation in order to achieve microbiological safety of milk. This is accomplished by optimizing the processes of bactofugation, ultrasound treatment (frequency 24 kHz and power 400 W) and slightly elevated temperatures, up to 55 °C, with the emphasis on the microbiological quality of milk, in accordance with legislation (somatic cell count in 1 mL must be ≤400 000). In bactofuged milk processed by high-power ultrasound, high inactivation of the total number of bacteria (from 1.61 to 1.77 log CFU/mL) was observed. The findings suggest that there is a possible application of new technologies in food processing as an effective replacement for thermal treatment; thus bactofugation in combination with high-power ultrasound could be an alternative to pasteurization.
